# Repeated prefrontal transcranial direct current stimulation improves behavioral regulation and modulates resting-state EEG microstate dynamics in young adults with problematic smartphone use: a randomized sham-controlled longitudinal study

**DOI:** 10.3389/fpsyt.2026.1928163

**Published:** 2026-10-19

**Authors:** Jun Chen, Jianming Cheng, Jinghua Teng, Hui Liu, Bo Yang, Yanxia Zhu, Enming Zhang, Fan Cheng, Yingying Zeng

**Affiliations:** 1 Cardiopulmonary Rehabilitation Center, Taihe Hospital, Hubei University of Medicine, Shiyan, Hubei, China; 2 Beijing Sport University, Beijing, China; 3 Department of Orthopaedic Rehabilitation, Taihe Hospital, Hubei University of Medicine, Shiyan, Hubei, China; 4 Department of Cardiology, Taihe Hospital, Hubei University of Medicine, Shiyan, Hubei, China; 5 Department of General Medical Services, Taihe Hospital, Hubei University of Medicine, Shiyan, Hubei, China; 6 Department of Radiology, Taihe Hospital, Hubei University of Medicine, Shiyan, Hubei, China

**Keywords:** digital behavioral addiction, EEG microstates, neuromodulation, problematic smartphone use, transcranial direct current stimulation

## Abstract

**Background:**

Problematic smartphone use (PSU) has emerged as an increasingly prevalent digital behavioral addiction among young adults and is associated with impaired executive control, excessive craving, and altered large-scale brain network organization. Although transcranial direct current stimulation (tDCS) has demonstrated therapeutic potential for addictive disorders, its neurophysiological mechanisms in PSU remain insufficiently understood. This study investigated whether repeated prefrontal tDCS improves behavioral outcomes and reorganizes resting-state electroencephalographic (EEG) microstate dynamics in young adults with PSU.

**Methods:**

Eighty participants meeting validated criteria for PSU were randomly assigned to receive active (n = 40) or sham (n = 40) tDCS. Active stimulation (2.0 mA, 20 min/session, three sessions/week for 12 weeks) targeted the left dorsolateral prefrontal cortex. Behavioral outcomes included objectively recorded smartphone use, Smartphone Addiction Scale–Short Version scores, ecological momentary assessment craving, and work and social functioning. Resting-state EEG was recorded under eyes-closed and eyes-open conditions before and after intervention. Canonical EEG microstate temporal characteristics were analyzed using mixed repeated-measures analysis with false discovery rate correction.

**Results:**

Compared with sham stimulation, active tDCS significantly reduced objectively measured smartphone use, craving intensity, and functional impairment while improving behavioral regulation. Neurophysiologically, active stimulation selectively reorganized resting-state brain dynamics. During eyes-closed recording, microstate C occurrence decreased whereas microstate D occurrence increased significantly. During eyes-open recording, both the occurrence and coverage of microstate B increased following active stimulation. No significant changes were observed in global explained variance, suggesting that repeated tDCS primarily modified the temporal organization rather than the spatial configuration of resting-state brain activity.

**Conclusions:**

Repeated prefrontal tDCS produced concurrent behavioral improvement and selective reorganization of resting-state EEG microstate dynamics in young adults with PSU. These findings provide preliminary evidence that repeated prefrontal tDCS is associated with concurrent behavioral changes and alterations in resting-state EEG microstate dynamics in young adults with PSU. Further confirmatory studies are needed to determine the clinical and mechanistic significance of these neurophysiological changes.

## 1 Introduction

Problematic smartphone use (PSU) has emerged as a behavioral pattern characterized by excessive and poorly controlled smartphone engagement associated with functional impairment, emotional dysregulation, and reduced self-regulatory capacity (1, 2). Increasing evidence suggests that PSU is associated with attentional dysfunction, impulsive decision-making, mood disturbances, and impaired academic and social functioning in young adults (3, 4). However, contemporary perspectives increasingly argue that excessive smartphone use should not be simplistically conceptualized as a classical substance addiction, but rather as a dimensional behavioral dysregulation phenotype involving altered cognitive control, reward-related processing, and maladaptive habitual behavior (5, 6). Understanding the neurophysiological mechanisms underlying PSU may therefore facilitate the development of scalable interventions targeting maladaptive digital-use behaviors.

Current interventions for PSU, including cognitive behavioral therapy, motivational approaches, and mindfulness-based interventions, may provide benefits for some individuals (7, 8). Nevertheless, sustained engagement, accessibility, and long-term adherence remain challenging, particularly among young adult populations characterized by high digital dependence and fluctuating treatment motivation. These limitations have contributed to increasing interest in adjunctive neuromodulatory approaches targeting neural systems involved in cognitive control and behavioral regulation. This rationale is particularly relevant to PSU, which has been associated with impaired inhibitory control and altered recruitment of prefrontal systems involved in cognitive control (9, 10). The dorsolateral prefrontal cortex (DLPFC) plays an important role in executive control and top-down regulation of motivational behavior and therefore represents a plausible neuromodulatory target for maladaptive patterns of smartphone use.

Transcranial direct current stimulation (tDCS) is a non-invasive neuromodulation technique in which weak direct currents delivered through scalp electrodes can modify cortical excitability (11). Repeated prefrontal tDCS has been investigated in substance-use and other addiction-related conditions, with systematic reviews and meta-analysis suggesting potential effects on craving and consumption-related outcomes, although findings vary across disorders and stimulation protocols (12, 13). Accordingly, targeting the DLPFC with tDCS may provide a mechanistically motivated approach for examining whether modulation of prefrontal control systems can improve behavioral regulation in individuals with PSU. Nevertheless, because direct therapeutic evidence in PSU remains limited, the present application of tDCS should be considered exploratory rather than an established treatment approach. Resting-state neuromodulation studies have increasingly focused on functional systems previously associated with executive control, salience processing, and internally directed cognition. However, direct network-level interpretations remain inferential, particularly when derived from scalp electrophysiological measures. In this context, electroencephalographic (EEG) microstate analysis provides a temporally sensitive approach for characterizing quasi-stable large-scale patterns of brain activity at the millisecond scale (14, 15). EEG microstates represent brief periods of relatively stable scalp topographies that are thought to reflect coordinated activity across distributed neural systems (16). Four canonical microstate classes, conventionally labeled A–D, are commonly identified during resting-state EEG and have been associated with different aspects of large-scale information processing (17). Of particular relevance to the present study, previous EEG–fMRI and source-localization studies have associated microstate B with visual and attention-related processing, microstate C with salience-related processing, and microstate D with attention and executive-control-related processes (18). These functional associations are not one-to-one mappings between individual microstate classes and specific brain networks and should therefore be interpreted cautiously. Compared with functional magnetic resonance imaging, EEG microstate analysis offers substantially higher temporal resolution, making it particularly suitable for examining rapid fluctuations in large-scale brain dynamics.

Altered microstate temporal dynamics have been reported across multiple psychiatric and neurobehavioral conditions, including schizophrenia, depression, anxiety disorders, autism spectrum disorder, and substance-use disorders (19–21). These findings support the potential utility of microstate analysis as a transdiagnostic neurophysiological marker of altered large-scale brain organization. Evidence directly examining tDCS in PSU remains scarce. However, studies in substance-use and related addictive or compulsive conditions provide a relevant neurobehavioral framework. Altered resting-state EEG microstate dynamics have been reported in both behavioral and substance-related addictive disorders, including internet gaming disorder and alcohol use disorder (21). These findings suggest that the temporal organization of large-scale resting-state activity may be sensitive to neurobehavioral alterations relevant to addictive behaviors. Neuromodulation studies in addiction further suggest that stimulation of the left DLPFC can be accompanied by alterations in resting-state EEG microstate dynamics; for example, microstate changes have been observed following intermittent theta-burst stimulation in individuals with heroin addiction (22). Complementary evidence from obsessive-compulsive disorder indicates that tDCS can also be accompanied by alterations in EEG microstate dynamics. Together, these findings provide an indirect, rather than disorder-specific, rationale for examining whether behavioral changes following prefrontal tDCS in PSU are accompanied by changes in resting-state microstate organization. To our knowledge, few studies have combined prefrontal tDCS with resting-state EEG microstate analysis in problematic smartphone use. We therefore conducted an exploratory sham-controlled study to investigate whether prefrontal tDCS would be associated with longitudinal behavioral changes and alterations in temporal microstate dynamics in university students with PSU. We hypothesized that, relative to sham stimulation, active prefrontal tDCS would be associated with greater reductions in objectively recorded smartphone-use duration and craving-related measures, together with improvement in functional outcomes. Given the limited prior literature linking tDCS to specific EEG microstate classes in PSU, we did not specify directional hypotheses for individual microstates. Instead, EEG microstate analysis were treated as exploratory, and we examined whether active stimulation was associated with changes in the occurrence, duration, coverage, or global explained variance (GEV) of canonical microstates under eyes-closed and eyes-open resting conditions.

## 2 Methods

### 2.1 Study design

This exploratory, randomized, sham-controlled neuromodulation study investigated longitudinal behavioral changes and resting-state electroencephalographic (EEG) microstate dynamics following prefrontal transcranial direct current stimulation (tDCS) in university students with problematic smartphone use (PSU). The study employed a parallel-group design with repeated behavioral and neurophysiological assessments conducted at baseline and after the intervention period, followed by longitudinal behavioral follow-up.

The study was not prospectively registered in a WHO/ICMJE-recognized public clinical-trial registry before enrollment of the first participant. The study protocol and analytic framework were subsequently archived on the Open Science Framework (OSF) prior to manuscript submission; this OSF record does not constitute prospective clinical-trial registration. Accordingly, the intervention-related findings are presented as exploratory and should be interpreted as preliminary and hypothesis-generating.

The study was approved by the Ethics Committee of Beijing Sport University (Approval No. 2022211H) and conducted in accordance with the Declaration of Helsinki. Written informed consent was obtained from all participants before enrollment.

The overall workflow of participant enrollment, intervention procedures, EEG acquisition, microstate analysis, and statistical processing is illustrated in **Figures 1** and **2**.

**Figure 1:**
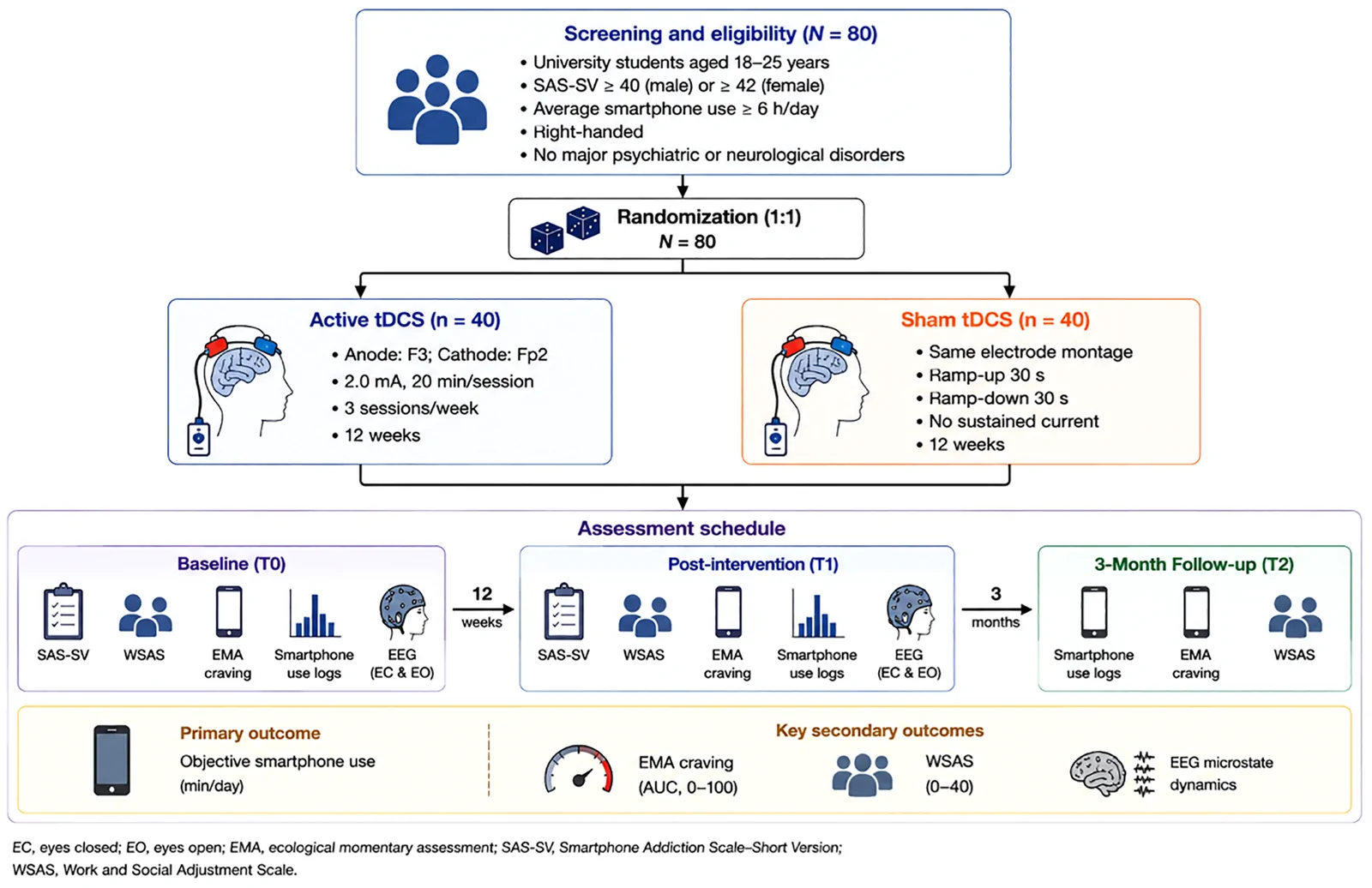
Study flow and assessment schedule.

**Figure 2:**
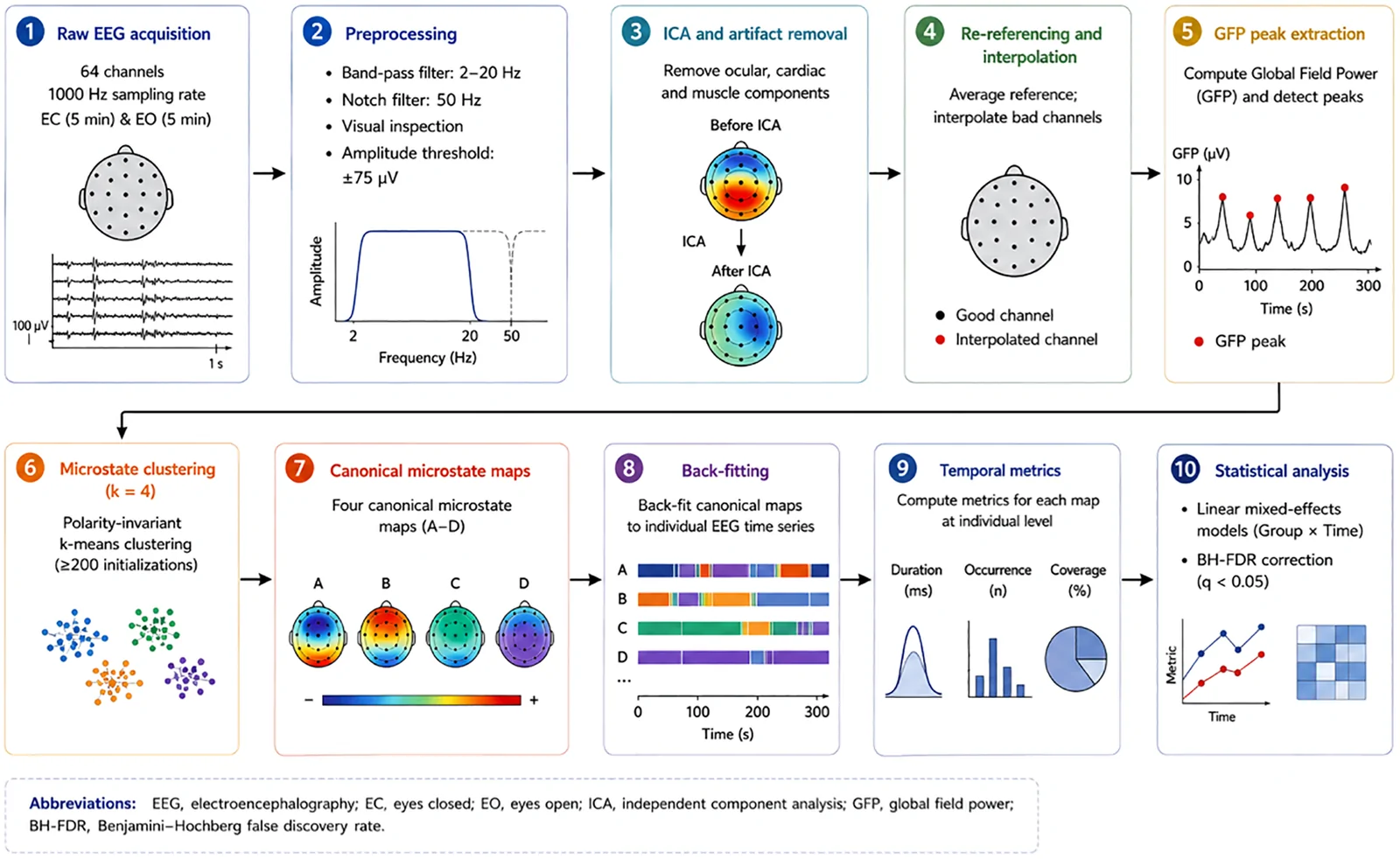
EEG preprocessing and microstate analysis pipeline.

### 2.2 Participants

Participants were recruited from university campuses through online advertisements and campus-based announcements between March 2024 and July 2024. Eligible participants were screened using the Smartphone Addiction Scale–Short Version (SAS-SV) and structured behavioral interviews focusing on excessive smartphone use patterns, functional impairment, and loss of behavioral control.

Participants were eligible if they:

1. were aged 18–25 years;
2. met screening criteria for elevated PSU-related behavioral characteristics (met elevated PSU screening criteria defined in the present study as SAS-SV scores ≥40 for males and ≥42 for females; these conservative study-specific thresholds were higher than the original SAS-SV validation cutoffs and were used to enrich the sample for individuals with more pronounced PSU-related characteristics.);
3. reported average smartphone use ≥6 h/day during the preceding month;
4. were right-handed;
5. had normal or corrected-to-normal vision.

Participants were excluded if they:

1. had a history of neurological or major psychiatric disorders;
2. had current substance-use disorders;
3. had previous exposure to tDCS;
4. were taking psychoactive medications;
5. had contraindications to non-invasive brain stimulation;
6. reported unstable sleep schedules or severe sleep deprivation during the assessment period.

Participants were not recruited based on formal psychiatric diagnostic criteria, but rather on elevated PSU-related behavioral characteristics and impaired self-regulatory smartphone use patterns.

Given the exploratory neurophysiological nature of the study and the limited prior literature combining tDCS with EEG microstate analysis in PSU, no formal *a priori* power calculation was performed. Sample size was guided by feasibility considerations and previous resting-state neuromodulation studies employing EEG microstate analysis (14, 19).

### 2.3 Randomization and blinding

Participants were randomly assigned in a 1:1 ratio to either the active tDCS group or the sham stimulation group using a computer-generated randomization sequence created by an investigator who was not involved in outcome assessment or data analysis. Allocation concealment was maintained using sequentially numbered opaque envelopes.

Participants and outcome assessors were blinded to treatment allocation throughout the study. The personnel administering tDCS were aware of the assigned stimulation condition because they were required to implement the active or sham stimulation protocol; however, they were not involved in behavioral outcome assessment or EEG data analysis. The sham procedure was designed to reproduce the transient scalp sensations associated with active stimulation through brief current delivery at the beginning and end of the session, thereby supporting participant blinding.

Blinding integrity was not formally evaluated using a prospectively standardized and retrievable assessment in either participants or outcome assessors. Because the personnel administering tDCS were aware of treatment allocation, provider blinding was not feasible.

### 2.4 tDCS protocol

tDCS was delivered using a battery-driven constant-current stimulator (NeuroConn GmbH, Germany) through saline-soaked sponge electrodes (5 × 5 cm). The left dorsolateral prefrontal cortex (DLPFC) was selected as the stimulation target because of its established involvement in executive control, inhibitory regulation, and top-down modulation of reward- and craving-related behavior, processes that are relevant to impaired behavioral regulation in problematic smartphone use (PSU). Previous studies in substance-use and related addictive behaviors have also targeted the DLPFC with tDCS and reported potential effects on craving, executive function, and consumption-related behavior. Accordingly, the anodal electrode was positioned over F3 according to the international 10–20 EEG system to target the left DLPFC, consistent with previous prefrontal tDCS studies in addictive disorders (23). while the cathodal electrode was positioned over the contralateral right supraorbital region (Fp2). This left-prefrontal/right-supraorbital configuration has been used in previous studies of prefrontal neuromodulation and addiction-related behavioral regulation (24).

Active stimulation was delivered at 2.0 mA for 20 min per session, with 30-s ramp-up and ramp-down periods. The choice of 2.0 mA and a 20-min stimulation duration was based on commonly used parameters in repeated prefrontal tDCS studies of addictive and craving-related behaviors, in which stimulation intensities of approximately 1.5–2.0 mA and session durations of approximately 20 min have been frequently employed (25). Participants received three sessions per week for 12 consecutive weeks. Because no established tDCS dosing schedule currently exists for PSU, the three-session-per-week, 12-week regimen was study-defined to provide repeated prefrontal stimulation over an extended intervention period while maintaining practical feasibility and participant tolerability; it should therefore not be interpreted as an established or optimized therapeutic dosing regimen for PSU.

For sham stimulation, the electrode montage was identical to that used in the active condition, but current was delivered only during the initial and final 30 s of the session to reproduce the transient cutaneous sensations associated with stimulation and thereby support participant blinding without providing sustained stimulation.

Participants were monitored throughout each stimulation session for common stimulation-related adverse effects, including itching, tingling, headache, dizziness, and discomfort. Adverse-effect monitoring was conducted as part of routine session safety surveillance; however, event-level data were not prospectively collected in a standardized format suitable for group-wise quantitative comparison.

### 2.5 Behavioral assessments

In addition to the primary behavioral outcomes, baseline affective symptoms, sleep quality, and substance-use characteristics were assessed to characterize the sample. Positive and negative affect were evaluated using the Positive and Negative Affect Schedule (PANAS) (26), a validated 20-item measure comprising separate Positive Affect and Negative Affect subscales, depressive symptoms using the PatientHealth Questionnaire-9 (PHQ-9), sleep quality using the Pittsburgh Sleep Quality Index (PSQI), alcohol use using the Alcohol Use Disorders Identification Test (AUDIT), and substance-use characteristics using the Drug Abuse Screening Test-10 (DAST-10). These measures were collected for descriptive purposes and were not included in the primary efficacy analysis.

#### 2.5.1 Objective smartphone use

The primary behavioral outcome was longitudinal change in objectively recorded smartphone use. Daily smartphone-use duration was continuously recorded using built-in operating-system screen-time monitoring applications. Average daily smartphone use was calculated for baseline, post-intervention, and follow-up periods.

#### 2.5.2 Smartphone addiction scale–short version

The Smartphone Addiction Scale–Short Version (SAS-SV) was used to assess PSU-related behavioral severity. The Smartphone Addiction Scale–Short Version (SAS-SV) is a 10-item self-report measure developed to assess problematic smartphone-use severity. The original validation study demonstrated high internal consistency and concurrent validity, with sex-specific screening cutoffs of 31 for males and 33 for females (27). The Chinese version has also demonstrated satisfactory internal consistency, test–retest reliability, and construct validity in adults. In the present study, the SAS-SV was used as a dimensional measure of PSU-related severity, while the more conservative study-specific thresholds described above were applied for participant screening.

#### 2.5.3 Work and social adjustment scale

Functional impairment associated with smartphone use was evaluated using the Work and Social Adjustment Scale (WSAS), a brief validated measure of impairment in work and social functioning (28).

#### 2.5.4 Ecological momentary assessment of craving

Daily craving for smartphone use was assessed using ecological momentary assessment (EMA) procedures. Participants rated craving intensity on a visual analog scale ranging from 0 (“no craving”) to 100 (“extreme craving”). Area-under-the-curve (AUC) values were calculated across repeated assessments.

#### 2.5.5 Exploratory durability outcome

An exploratory behavioral durability outcome reflecting sustained return to elevated smartphone use was additionally examined using individualized thresholds derived from baseline smartphone-use patterns. This measure was considered exploratory and was not treated as a formal clinical endpoint.

### 2.6 EEG acquisition

Resting-state EEG data were acquired using a 64-channel Neuroscan SynAmps2 amplifier system (Compumedics Neuroscan, USA) positioned according to the international 10–20 system. Signals were sampled at 1000 Hz with impedances maintained below 5 kΩ.

Participants completed both eyes-closed (EC) and eyes-open (EO) resting-state recordings in a quiet, dimly lit, electrically shielded room. Each condition lasted approximately 5 min. During EO recordings, participants were instructed to maintain visual fixation on a centrally presented cross.

Horizontal electrooculogram (HEOG) signals were recorded from electrodes positioned at the outer canthi, while vertical electrooculogram (VEOG) signals were recorded above and below the right eye to facilitate artifact correction.

### 2.7 EEG preprocessing

EEG preprocessing was performed using EEGLAB (version 2023.0; 29) implemented in MATLAB R2023b (MathWorks, Natick, MA, USA).

Continuous EEG data were band-pass filtered between 2 and 20 Hz to reduce slow drifts and high-frequency noise. Gross artifacts were visually inspected and removed manually. Independent component analysis (ICA) was subsequently performed to identify and remove ocular and muscle-related artifacts.

Artifact-contaminated segments exceeding ±75 μV were rejected automatically, followed by visual inspection to ensure signal quality. Bad channels were interpolated using spherical spline interpolation, and EEG data were re-referenced to the average reference.

Only artifact-free data segments were retained for subsequent microstate analysis.

### 2.8 EEG microstate analysis

EEG microstate analysis was conducted using the Microstate EEGlab Toolbox (30) implemented in MATLAB. Global field power (GFP) peaks were extracted from the preprocessed resting-state EEG data because these time points provide a high signal-to-noise ratio and high topographic stability.

K-means clustering with polarity invariance was applied to GFP peak maps using at least 200 random initializations to improve clustering stability. Consistent with prior resting-state microstate literature, a four-class solution (microstates A–D) was selected based on cross-validation criteria and comparability with canonical microstate configurations reported in previous studies.

Group-level template maps were subsequently back-fitted to individual EEG recordings. Temporal parameters including mean duration (ms), occurrence (Hz), and coverage (%) were calculated for each microstate class. Mean duration represented the average time for which a given microstate remained continuously active, occurrence represented the average number of appearances of that microstate per second, and coverage represented the proportion of total recording time occupied by that microstate. Global explained variance (GEV) represented the proportion of EEG topographic variance explained by the fitted microstate model (14).

Because scalp-level microstates provide indirect markers of large-scale brain dynamics, network-level interpretations were considered inferential. Accordingly, EEG microstate measures were treated as exploratory neurophysiological outcomes.

### 2.9 Statistical analysis

Statistical analysis were conducted using SPSS version 27.0 (IBM Corp., USA) and R version 4.3.1.

Baseline demographic and behavioral variables were compared between groups using independent-samples t-tests or chi-square tests where appropriate.

Longitudinal behavioral outcomes and EEG microstate parameters were analyzed using repeated-measures analysis of variance (ANOVA) with Group (active vs. sham) as the between-subject factor and Time (baseline vs. post-intervention) as the within-subject factor. Greenhouse–Geisser correction was applied where sphericity assumptions were violated.

Primary behavioral nalysis focused on objectively recorded smartphone-use duration. EEG microstate analysis were considered exploratory because of the limited prior literature examining microstate alterations following tDCS in PSU.

To reduce inflation of Type I error, Benjamini–Hochberg false discovery rate (BH-FDR) correction was applied within predefined families of EEG outcomes.

Exploratory time-to-event analyses were used to evaluate the durability outcome during the 90-day follow-up period. Between-group differences in time to sustained return to elevated smartphone-use behavior were summarized using Cox proportional-hazards models, with effect estimates reported as hazard ratios (HRs) and 95% confidence intervals (CIs). Restricted mean survival time (RMST) over 90 days was additionally calculated to quantify between-group differences in event-free time. Sensitivity analyses were performed using alternative operational definitions of the behavioral threshold to evaluate the robustness of the findings. Because these analyses concerned an exploratory, non-validated behavioral durability outcome, the resulting estimates were interpreted as hypothesis-generating.

Effect sizes were reported as partial eta squared (ηp²), and statistical significance was defined as p < 0.05 (two-tailed).

## 3 Results

### 3.1 Baseline characteristics

A total of 80 participants were included (tDCS**/**sham = 40/40). The two groups were balanced at baseline in age, sex, years of education, SAS-SV total score, mean daily screen time, PANAS-PA/NA, PSQI, PHQ-9, AUDIT and DAST-10 (all *p* >.10). Overall, the sample had a mean age 20 years, 50% female, 15 years of education, SAS-SV 46, and 8 h/day smartphone use; sleep and mood indices were mostly in the mild–moderate range. Detailed demographics and baseline measures are shown in **Table 1**.

**Table 1:** Demographic and baseline characteristics of the sample.

Characteristic	Total(N = 80)	tDCS(n=40)	Sham(n=40)	p-value
Age,years	20.2 ± 1.5	20.3 ± 1.6	20.1 ± 1.4	0.56
Female, n (%)	40 (50.0)	20 (50.0)	20 (50.0)	1.00
Years of education	15.1 ± 1.4	15.2 ± 1.4	15.0 ± 1.5	0.54
SAS-SV total (10–60)	45.8 ± 4.9	46.0 ± 5.0	45.6 ± 4.9	0.68
Daily screen time, h/day	8.1 ± 1.6	8.2 ± 1.6	8.0 ± 1.5	0.48
PANAS-PA (10–50)	27.1 ± 5.9	27.0 ± 6.0	27.2 ± 5.9	0.90
PANAS-NA (10–50)	18.2 ± 5.1	18.1 ± 5.2	18.3 ± 5.1	0.84
PSQI (0–21)	6.1 ± 2.1	6.0 ± 2.1	6.2 ± 2.0	0.63
AUDIT (0–40)	6.2 ± 3.1	6.1 ± 3.2	6.3 ± 3.0	0.78
PHQ-9 (0–27)	8.0 ± 3.9	7.9 ± 4.0	8.1 ± 3.8	0.79

Participants met the SAS-SV threshold for inclusion. PHQ-9 and PSQI were used for exclusion/description only and were not included as covariates in primary models.

### 3.2 EEG microstates: eyes-closed

Under the EC condition, microstate C occurrence showed a significant Group × Time interaction (ΔΔ ≈ −1.7 Hz, 95% CI −3.0 to −0.5, p = .006, partial η² ≈.09). This interaction reflected a greater decrease from baseline to post-intervention in the active-tDCS group than in the sham group. In contrast, microstate D occurrence showed a significant Group × Time interaction (ΔΔ ≈ +1.0 Hz, 95% CI 0.2–1.8, p = .014, partial η² ≈.07), reflecting a greater increase from baseline to post-intervention in the active-tDCS group relative to the sham group. No other EC metrics demonstrated significant Group × Time interactions. See **Table 2** and **Figure 3**.

**Table 2:** EEG microstates (eyes-closed): group×time mixed ANOVA (occurrence, coverage and duration).

Outcome(EC)	Pre mean ± SD	Post mean ± SD	ΔΔ(95%CI)	p	Partial η²(95%CI)
MicrostateC occurrence(Hz)	tDCS11.2 ± 3.8/Sham11.3 ± 3.9	tDCS9.1 ± 3.9**/**Sham10.9 ± 3.8	−1.7(−3.0to−0.5)	0.006	0.086 (0.018–0.168)
MicrostateD occurrence(Hz)	tDCS3.9 ± 2.6/Sham4.0 ± 2.7	tDCS5.0 ± 2.7**/**Sham4.1 ± 2.6	+1.0(+0.2to+1.8)	0.014	0.074 (0.011–0.154)
MicrostateB occurrence(Hz)	tDCS7.4 ± 3.2/Sham7.3 ± 3.1	tDCS7.2 ± 3.3/Sham7.1 ± 3.2	+0.0(−0.9to+0.9)	0.975	0.000 (0.000–0.010)
GEV(%)	tDCS55.8 ± 8.2/Sham56.0 ± 8.1	tDCS56.4 ± 8.6/Sham54.9 ± 8.4	+1.3(−1.2to+3.7)	0.311	0.012 (0.000–0.059)

ΔΔ = (tDCS: Post − Pre) − (Sham: Post − Pre), derived from the Time×Group interaction; positive values indicate an increase in the tDCS grouprelative to sham, and negative values indicate a relative decrease.

**Figure 3:**
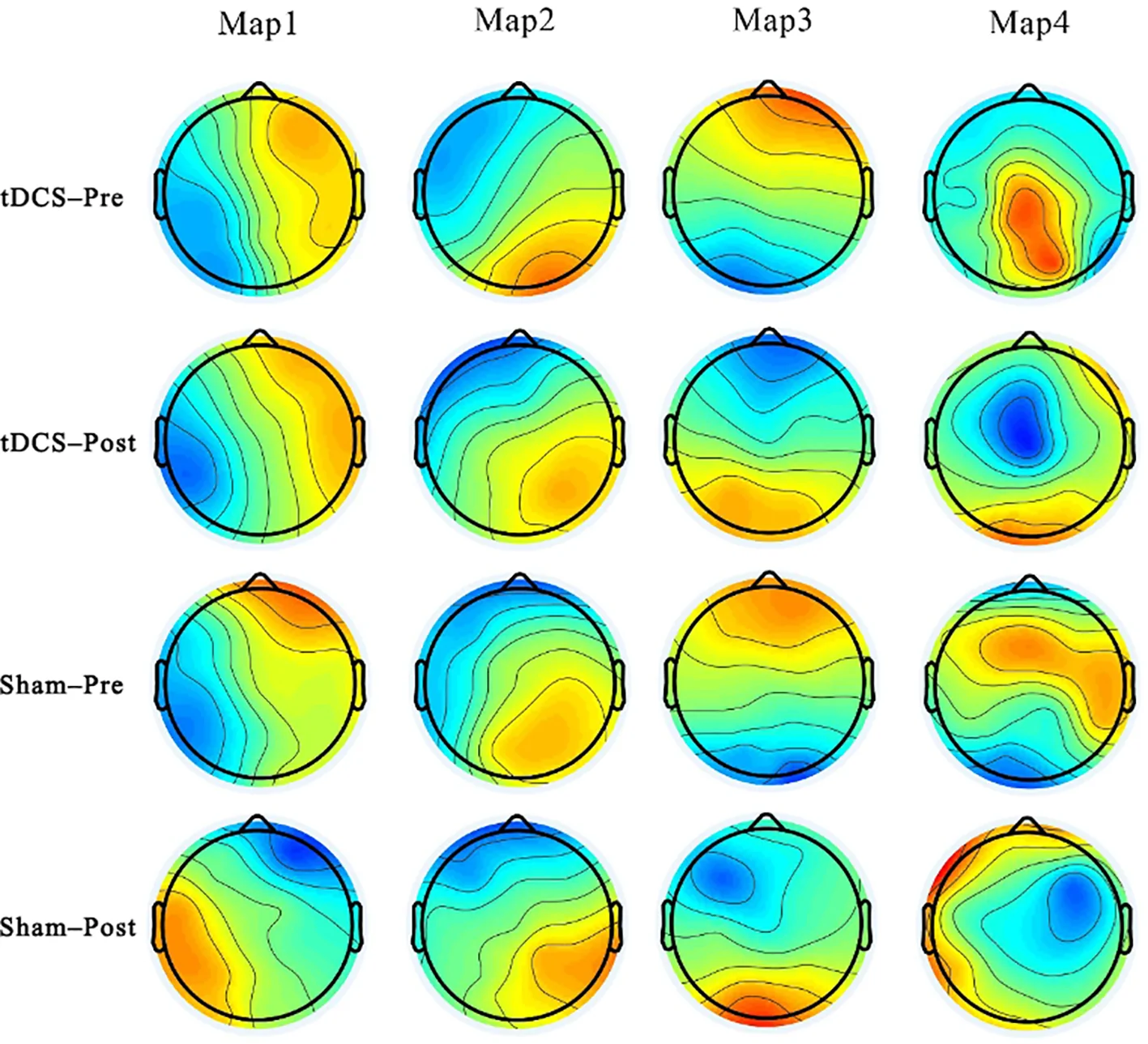
Eyes-closed (EC) EEG microstate topographies: pre- and post-intervention maps in the tDCS and sham groups. Orientation note: Scalp view from above (nose up, left ear to the left). Red and blue denote opposite polarities of the same potential map and are color labels without physiological valence.

### 3.3 EEG microstates: eyes-open

Under the EO condition, microstate B occurrence showed a significant Group × Time interaction (ΔΔ ≈ +1.6 Hz, 95% CI 0.3–2.8, p = .017, partial η² ≈.07), reflecting a greater increase from baseline to post-intervention in the active-tDCS group relative to the sham group. Microstate B coverage showed a similar pattern (ΔΔ ≈ +3.4%, 95% CI 0.6–6.2, p = .018), with a greater increase in the active-tDCS group relative to sham. No other EO metrics exhibited significant Group × Time interactions. See **Table 3** and **Figure 4**.

**Table 3:** EEG microstates (eyes-open, EO): Group×Time mixed ANOVA.

Outcome (EO)	Pre mean ± SD	Post mean ± SD	ΔΔ (95% CI)	p	Partial η² (95% CI)
Microstate-B occurrence (Hz) *(primary)*	tDCS 8.9 ± 3.0/Sham 8.8 ± 3.1	tDCS 10.2 ± 3.1**/**Sham 8.6 ± 3.0	+1.6 (+0.3 to +2.8)	0.017	0.070 (0.010–0.150)
Microstate-B coverage (%) *(key secondary)*	tDCS 29.6 ± 11.8/Sham 28.9 ± 11.7	tDCS 33.7 ± 12.1/Sham 27.8 ± 11.5	+3.4 (+0.6 to +6.2)	0.018	0.069 (0.009–0.148)
Microstate-A duration (ms)	tDCS 26.0 ± 4.8/Sham 25.7 ± 4.9	tDCS 26.6 ± 4.5/Sham 26.0 ± 4.7	+0.3 (−0.8 to +1.4)	0.585	0.004 (0.000–0.040)
Microstate-C occurrence (Hz)	tDCS 9.3 ± 4.6/Sham 9.2 ± 4.7	tDCS 8.0 ± 4.9/Sham 8.5 ± 4.8	−0.6 (−1.8 to +0.6)	0.314	0.012 (0.000–0.060)
Global explained variance, GEV (%)	tDCS 52.4 ± 8.7/Sham 51.6 ± 8.9	tDCS 54.1 ± 9.0/Sham 50.8 ± 8.6	+2.1 (−0.5 to +4.7)	0.114	0.028 (0.000–0.090)

ΔΔ = (tDCS: Post − Pre) − (Sham: Post − Pre).

**Figure 4:**
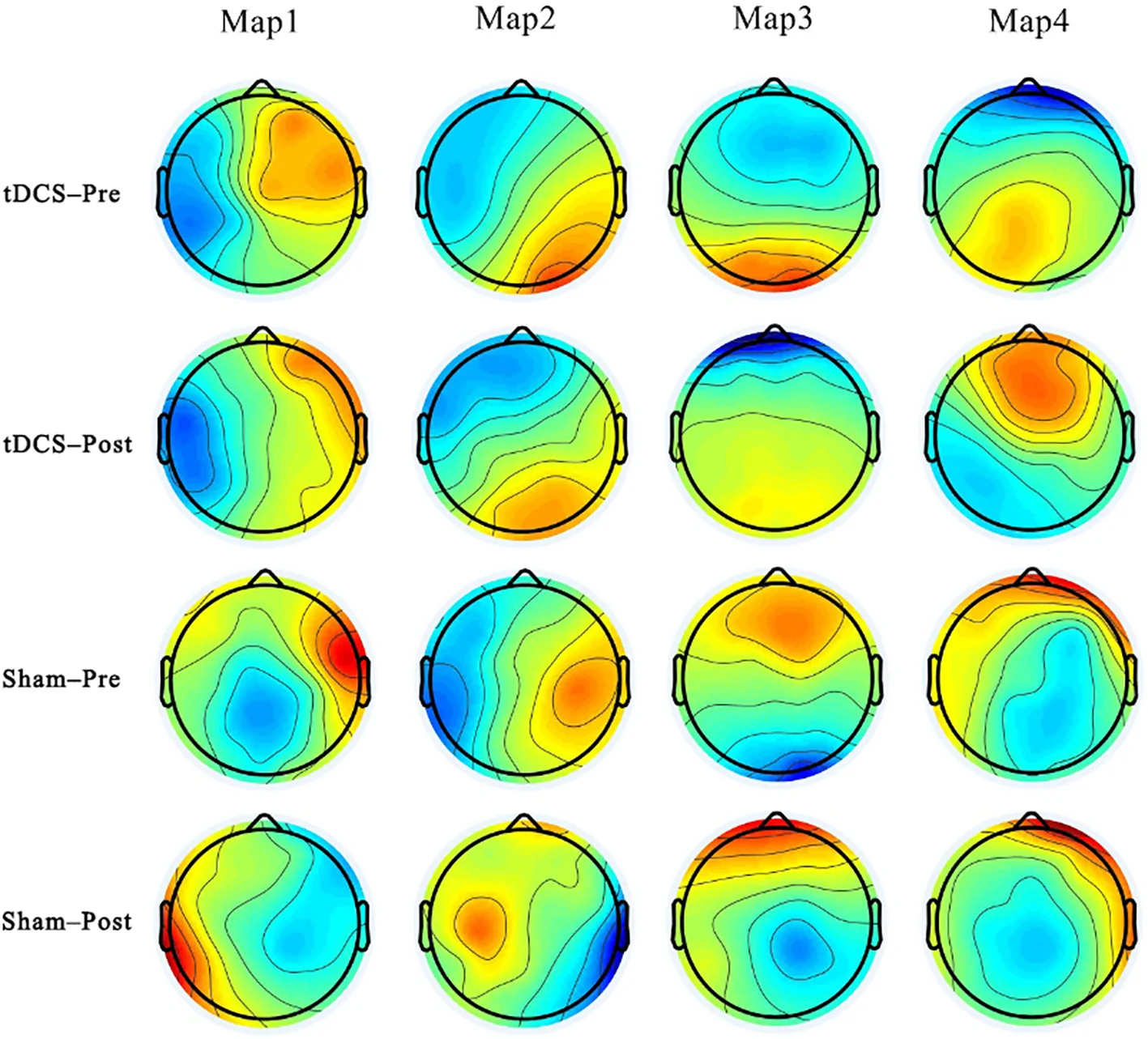
Eyes-open (EO) EEG microstate topographies: pre- and post-intervention maps in the tDCS and sham groups. Orientation note: Scalp view from above (nose up, left ear to the left). Red and blue indicate opposite polarities of the same potential map and are arbitrary color labels without physiological meaning.

### 3.4 Follow-up and behavioral durability outcome

Exploratory follow-up analysis examined maintenance of reduced smartphone-use behavior during the 90-day observation period (**Table 4**). Because participants were not enrolled following formal clinical remission, the endpoint was operationalized as sustained return to elevated smartphone-use behavior rather than sustained return to elevated smartphone-use behavior in the classical substance-use framework.

**Table 4:** Exploratory durability outcome during follow-up.

Measure	Active tDCS	Sham	Between-group
1-month cumulative event rate (%)	18%	27%	Risk difference −9%(95%CI−20, 2)
3-month cumulative event rate (%)	31%	47%	Risk difference −16% (95% CI −31, −1)
Median time-to-event (days)	Not reached	38	–
Cox HR (Active vs Sham)	–	–	0.62 (95% CI 0.41–0.93), p=0.021
RMST difference (90 days)	–	–	+6.2 days (95% CI 0.8–11.5)

CI, confidence interval; HR, hazard ratio; RMST, restricted mean survival time. The event was defined as sustained return to elevated smartphone-use behavior and should be interpreted as an exploratory behavioral durability outcome rather than a validated clinical endpoint.

Across follow-up assessments, the cumulative event rate remained lower in the active stimulation group than in the sham group, suggesting modestly improved behavioral durability following active prefrontal stimulation. Time-to-event analysis demonstrated comparable directional patterns, with active stimulation associated with a lower likelihood of sustained return to elevated smartphone-use behavior across the follow-up interval.

Restricted mean survival time analysis further suggested prolonged maintenance of reduced smartphone-use behavior in participants receiving active stimulation. Sensitivity analysis using alternative behavioral thresholds demonstrated consistent directional findings (see **Supplementary Table S1**). However, because this endpoint reflected behavioral persistence rather than a validated clinical outcome, these findings should be interpreted cautiously and considered exploratory.

### 3.4 Behavioral and functional follow-up

Relative to sham stimulation, active tDCS was associated with lower objectively recorded daily smartphone-use duration across follow-up assessments (**Table 5**). Between-group differences were most pronounced immediately after intervention and gradually attenuated over time, although directional separation between groups remained evident at 3 months.

**Table 5:** Longitudinal behavioral and functional outcomes.

Outcome	Post difference (95%CI)	p	M1 difference (95%CI)	p	M3 difference (95%CI)	p
Mean daily screen time (min/day)	−44 (−68,−20)	0.0004	−36 (−60,−12)	0.004	−24 (−48,−0.4)	0.046
EMA craving AUC (0–100)	−9.2 (−14.1,−4.3)	0.0004	−7.1 (−12.0,−2.2)	0.005	−4.8 (−9.9,0.3)	0.066
WSAS total score (0–40)	−3.2 (−5.3,−1.0)	0.004	−2.6 (−4.8,−0.4)	0.021	−1.9 (−4.1,0.2)	0.078
TLFB-28 mean daily screen time (min/day)	−38 (−61,−15)	0.001	−30 (−52,−8)	0.008	−18 (−41,4)	0.108

Values represent between-group differences (active stimulation − sham stimulation). Negative values favor active stimulation. TLFB-28, 28-day Timeline Follow-Back; EMA, ecological momentary assessment; WSAS, Work and Social Adjustment Scale.

EMA craving AUC and WSAS scores demonstrated similar early improvements following active stimulation. Several behavioral outcomes no longer remained statistically significant at 3 months, suggesting partial attenuation of treatment-related effects over follow-up.

TLFB-28 estimates demonstrated comparable directional patterns with objective smartphone-use logs across assessment time points.

Behavioral trajectories across the intervention and follow-up periods are illustrated in **Figure 5**. Relative to sham stimulation, participants receiving active tDCS demonstrated greater reductions in objectively recorded smartphone-use duration immediately after intervention, with partial attenuation of effects during follow-up. Similar temporal patterns were observed for EMA craving burden and WSAS scores, indicating concurrent improvements in subjective craving and functional impairment. Across outcomes, the largest between-group differences emerged at the post-intervention assessment and gradually diminished over time, although directional separation between groups remained evident at 3 months.

**Figure 5:**
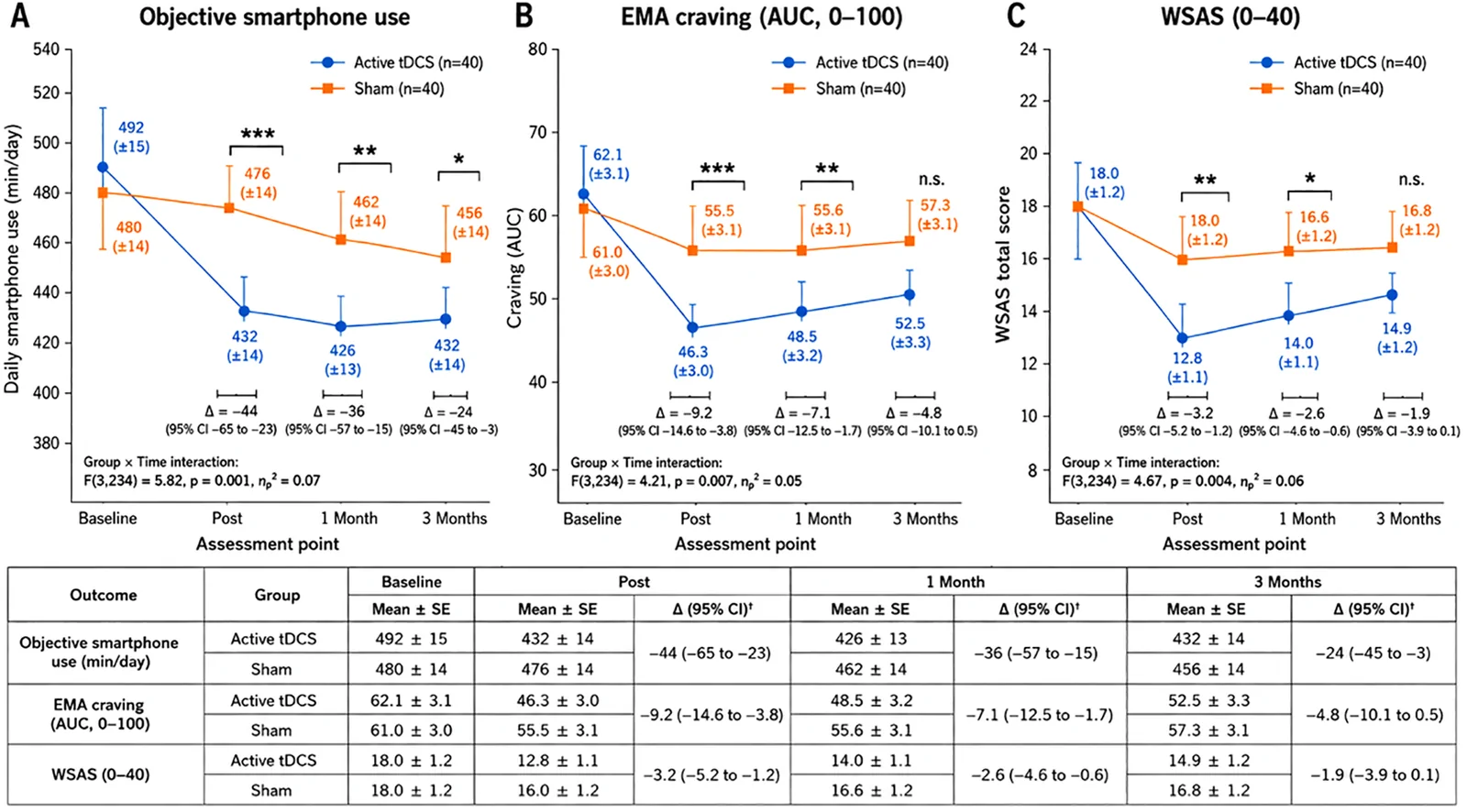
Longitudinal behavioral trajectories following prefrontal transcranial direct current stimulation in young adults with problematic smartphone use. **(A)** Objectively recorded smartphone-use duration. **(B)** Ecological momentary assessment (EMA) craving burden. **(C)** Work and Social Adjustment Scale (WSAS) scores.

Values are presented as mean ± SE. Error bars indicate standard errors. Δ values represent between-group differences (active tDCS − sham stimulation) at each follow-up assessment. Active stimulation was associated with greater reductions in smartphone-use duration, craving burden, and functional impairment compared with sham stimulation. Group × Time interaction statistics are shown within each panel.

## 4 Discussion

The present study suggests that active prefrontal tDCS may influence both smartphone-use behavior and resting-state EEG microstate temporal dynamics in individuals with problematic smartphone use (PSU). Specifically, active stimulation was associated with reduced EC microstate C occurrence, increased EC microstate D occurrence, and enhanced EO microstate B expression, whereas no significant alterations were observed for global explained variance or overall scalp topographies. These findings indicate that the principal effects of stimulation primarily involved temporal reorganization of resting-state activity rather than large-scale spatial restructuring of canonical microstate configurations (31, 32). Importantly, the behavioral trajectories shown in **Figure 5** paralleled the observed alterations in EEG microstate dynamics, suggesting that changes in large-scale brain-state organization may accompany improvements in behavioral regulation following prefrontal neuromodulation.

Our findings are broadly consistent with recent evidence showing that tDCS-induced clinical improvement may be accompanied by measurable alterations in EEG microstate dynamics in obsessive-compulsive disorder, supporting the potential utility of microstates as neurophysiological markers of treatment-related brain-state reorganization (33).

Recent conceptual and neurobehavioral models increasingly characterize PSU as a multidimensional behavioral dysregulation phenotype involving impaired salience attribution, reduced inhibitory control, maladaptive reward processing, and attentional dysregulation (34–37).Neuroimaging studies have further demonstrated altered functional connectivity and disrupted large-scale network organization in individuals with excessive smartphone or internet-related behaviors (38–40). Within this framework, the present microstate findings may reflect altered temporal coordination of large-scale resting-state neural activity following active prefrontal stimulation.

Previous simultaneous EEG–fMRI and source-localization studies have associated canonical EEG microstates with large-scale resting-state neural systems, with microstates C and D showing associations with brain regions implicated in salience-related processing and externally directed attentional or executive processes. However, these associations should not be interpreted as one-to-one correspondence between individual EEG microstates and specific functional brain networks. In the present study, the relative decrease in EC microstate C occurrence and increase in EC microstate D occurrence following active tDCS may therefore reflect altered temporal engagement of processes related to salience processing, attention, and executive regulation (18). This interpretation remains tentative and does not establish a specific neural mechanism (15, 20, 41). More recent psychiatric microstate studies have suggested that abnormalities in microstate temporal dynamics may reflect transdiagnostic disturbances in cognitive integration and large-scale neural coordination (32). In the present study, reduced EC microstate C occurrence alongside increased EC microstate D occurrence may tentatively indicate reduced salience-driven processing accompanied by relative enhancement of executive or attentional regulation following active stimulation.

Importantly, these interpretations remain indirect because EEG microstates cannot be directly equated with specific functional networks. Recent evidence from methamphetamine use disorder and obsessive-compulsive disorder likewise demonstrated distinct abnormalities in EEG microstate temporal dynamics, supporting the notion that disrupted large-scale brain-network coordination may represent a transdiagnostic feature across compulsive and addictive conditions (42).

The observed EO increase in microstate B expression may also be relevant. Although the functional significance of microstate B remains less consistently characterized than that of microstates C and D, previous studies have linked microstate B to visual and attentional processing (19, 43). Given that excessive smartphone use is strongly visually driven and continuously reinforced through externally oriented attentional engagement, modulation of EO microstate B may reflect altered processing of externally directed attentional states after prefrontal neuromodulation. However, this interpretation should remain cautious and requires validation in multimodal imaging paradigms.

The behavioral findings demonstrated a comparable temporal pattern. Active stimulation was associated with reductions in objectively recorded smartphone-use duration, craving-related burden, and functional impairment, with the strongest effects emerging immediately after intervention and gradually attenuating during follow-up. This attenuation pattern is broadly consistent with recent neuromodulation studies reporting predominantly short- to medium-term behavioral effects following repeated prefrontal stimulation across compulsive and addictive behaviors (44–47). Notably, the present findings do not suggest that tDCS “treats” smartphone addiction in a categorical sense. Rather, they support the possibility that prefrontal stimulation may transiently facilitate behavioral regulation and inhibitory control in individuals exhibiting elevated PSU characteristics.

Exploratory follow-up analysis further suggested partial maintenance of treatment-related behavioral improvement after completion of the intervention period. Although the durability findings appeared less pronounced than the immediate post-intervention effects, directional group differences remained evident throughout follow-up analysis. These findings align with recent literature emphasizing that behavioral improvements following non-invasive neuromodulation may diminish over time in the absence of continued reinforcement or adjunctive behavioral intervention (48, 49). Nevertheless, the persistence of directional separation between groups raises the possibility that prefrontal neuromodulation may exert short-term stabilizing effects on behavioral regulation in PSU.

The present study additionally contributes to the growing literature examining neurophysiological correlates of digital-use-related behavioral dysregulation. Most previous PSU neuromodulation studies have primarily focused on behavioral or questionnaire-based outcomes (50), whereas relatively few investigations have explored large-scale electrophysiological temporal dynamics following intervention. By integrating resting-state EEG microstate analysis with longitudinal behavioral follow-up, the current findings provide preliminary evidence that behavioral improvement following prefrontal stimulation may occur alongside measurable alterations in large-scale temporal brain dynamics.

Several limitations should be acknowledged. First, the study was exploratory in nature and included a relatively modest sample size, which may limit statistical stability and generalizability. The study was also not designed or powered to identify baseline predictors of treatment response; larger, adequately powered studies are needed to determine whether baseline clinical, behavioral, or neurophysiological characteristics can reliably identify individuals who are more likely to benefit from tDCS. Second, although participants and outcome assessors were blinded to treatment allocation, blinding integrity was not formally evaluated using a prospectively standardized and retrievable assessment. In addition, the personnel administering tDCS were aware of treatment allocation because of the stimulation procedures. Although treatment administration and outcome assessment were separated, potential performance bias related to provider awareness cannot be completely excluded. Third, EEG microstates provide indirect measures of large-scale neural dynamics and cannot be interpreted as definitive markers of specific functional networks or causal neural mechanisms. Fourth, the follow-up endpoint was operationalized as sustained return to elevated smartphone-use behavior and should therefore be interpreted as an exploratory behavioral outcome rather than a validated clinical endpoint. Fifth, although behavioral and neurophysiological changes occurred in parallel, causal mediation between microstate alterations and behavioral improvement was not established. Finally, the absence of multimodal neuroimaging data limits mechanistic interpretation of the observed electrophysiological findings.

Future studies combining EEG microstate analysis with functional neuroimaging, computational modeling, ecological behavioral tracking, and longer-term longitudinal follow-up may help clarify the neurophysiological significance and clinical relevance of prefrontal neuromodulation in PSU (51–53).

## 5 Conclusion

Repeated prefrontal tDCS was associated with temporal reorganization of resting-state EEG microstate dynamics, accompanied by parallel improvements in smartphone-use behavior. These findings provide preliminary evidence that behavioral regulation in PSU may be associated with measurable changes in large-scale brain-state organization.

## Data Availability

The raw data supporting the conclusions of this article will be made available by the authors, without undue reservation.

## Supplementary material

Supplementary Table 1 Sensitivity analyses for behavioral threshold definition (Cox HR and RMST difference within 90 days).
