# Supplementary material for: Repeated prefrontal transcranial direct current stimulation improves behavioral regulation and modulates resting-state EEG microstate dynamics in young adults with problematic smartphone use: a randomized sham-controlled longitudinal study
Source: Front Psychiatry. 2026 Oct 19;17:1928163. doi: 10.3389/fpsyt.2026.1928163 (PMC13725176; doi:10.3389/fpsyt.2026.1928163)
Supplement: Supplementary file 1 [file Table1.docx]

**Supplementary Material**

**Supplementary Table S1. Sensitivity analyses for behavioral threshold definition (Cox HR and RMST difference within 90 days)**

| **Alternative behavioral threshold definition** | **Cox HR (95% CI) p** | **RMST Δ (90 days, 95% CI)** | **Direction vs main analysis** |
| --- | --- | --- | --- |
| Main analysis: Individualized threshold T = baseline × (1 − 30%); > T for ≥2 days or ≥50% in 14 days | 0.62 (0.41–0.93) 0.021 | +6.2 (0.8 to 11.5) days | Reference |
| Absolute threshold: ≥ 180 min/day for ≥2 days | 0.65 (0.43–0.98) 0.037 | +5.8 (0.6 to 11.0) days | Consistent |
| Proportional threshold: ≥ 20% above baseline for ≥2 days | 0.60 (0.39–0.91) 0.025 | +6.5 (1.2 to 11.8) days | Consistent |
| Stricter persistence: > T for ≥3 days or ≥60% in 14 days | 0.63 (0.40–0.97) 0.033 | +6.0 (0.5 to 11.2) days | Consistent |
| No moving average: daily value > T for ≥2 days | 0.66 (0.44–1.00) 0.048 | +5.5 (0.4 to 10.7) days | Consistent |
